# Lamotrigine induced toxic epidermal necrolysis: A case report

**DOI:** 10.1016/j.amsu.2020.11.036

**Published:** 2020-11-17

**Authors:** Kiran Kumar KC, Trishant Limbu, Shirish Shakti Maskay, Anil Bhasima, Subhash Prasad Acharya

**Affiliations:** a Department of Critical Care Medicine, Grande International Hospital, Kathmandu, Nepal; b Department of Anaesthesiology, Institute of Medicine, Maharajgunj, Kathmandu, Nepal

**Keywords:** Lamotrigine, Toxic epidermal necrolysis, Cutaneous adverse reaction

## Abstract

**Introduction:**

A wide spectrum of cutaneous adverse reactions ranging from simple maculopapular rashes to more severe and life-threatening reactions like Stevens-Johnson syndrome (SJS) and toxic epidermal necrolysis(TEN) have been described after exposure to many antiepileptic drugs. Although the adverse effect following lamotrigine has been reported after a low initial dosage, the risk of developing TEN is relatively rare.

**Case report:**

We present a 23-year-old female, 6 months post-partum, a case of complex partial seizure, who developed TEN after 14 days of monotherapy with lamotrigine. She was put on steroids and other supportive management. After a tempestuous course of 9 days in ICU, she made an eventful recovery.

**Discussion:**

Lamotrigine, a chemically different newer antiepileptic, if rapidly titrated and used in conjunction with valproate can cause exfoliative dermatitis-like TEN, but at lower doses and as a monotherapy, female, post-partum, probably due to hormonal factors and strong association between HLA-B*1502 and AED (Antiepileptic drug)-induced SJS/TEN in patients of Asian ethnicity could be other contributing cause. Also, lesser use of lamotrigine in developing nations might have led to a lesser incidence of serious cutaneous adverse reactions. The SCORTEN (Severity-of-illness score for toxic epidermal necrolysis) is the most widely used system to standardize the evaluation of risk and prognosis in patients with TEN.

**Conclusion:**

Though rare but TEN can occur following lamotrigine monotherapy. Prompt diagnosis, withdrawal of offending agent, and timely proper supportive care might help in lowering the mortality.

## Highlights

• Severe exfoliative dermatitis like SJS and TEN are less reported with lamotrigine as a monotherapy compared to aromatic anticonvulsants.
• The severe adverse cutaneous reaction following lamotrigine use may be overlooked as it is not used that frequently in developing nation.
• Prompt diagnosis, withdrawal of offending agent and proper supportive care can help prevent mortality in such case.

## Introduction

Toxic epidermal necrolysis (TEN), a rare immune-mediated life-threatening reaction, is a severe form of Stevens-Johnson syndrome (SJS), in which involvement of body surface area (BSA) is more than 30% [1]. Incidence of TEN is 0.4–1.2 cases per million population per year [2] in which drugs account for the majority of cases. Beside Antibiotics and NSAIDS, Antiepileptics are common drugs associated with SJS/TEN [3]. Among Antiepileptic, Phenytoin and Carbamazepine are associated with a more severe cutaneous reaction [4]. Lamotrigine, an antiepileptic and a mood stabilizer can also cause anticonvulsant hypersensitivity syndrome clinically manifesting as severe maculopapular exanthema, fever, and lymphadenopathy. It can also cause hepatic, renal, hematologic, and pulmonary impairment [5]. Lamotrigine related simple maculopapular exanthemas are observed in up to 10% of users and mostly observed within the first eight weeks of treatment. However, SJS/TEN are noted in certain users with over-rapid titration when initiating therapy and concomitant use of valproate [6,7]. Here we report a case of lamotrigine induced TEN in a 23-year-old female, 6 months postpartum with a lower dose, and on monotherapy.

## Case report

A 23-year-old woman and 6 months postpartum, a known case of complex partial seizure for past one year under oxcarbazepine was switched to lamotrigine 50 mg two weeks back after she complained of diplopia and visual disturbances. Nearly two weeks after initiation of therapy, she started complaining of difficultly in swallowing, burning micturition, burning sensation in eyes, and developed oral ulcer and generalized purpuric rashes. She was initially admitted to the ward but few days later was shifted to ICU for monitoring and isolation because of the progression of skin lesion. Her SCORTEN score [8] was 2. During admission in ICU, the rashes had involved more than 30% of body surface area along with epidermal necrosis, and Nikolsky's sign was positive. The Naranjo Algorithm or Adverse Drug Reaction Probability Scale was used to assess the casualty analysis and it was found “probable” with lamotrigine [9]. The diagnosis of lamotrigine induced TEN was made. Her vitals were stable. Offending drug was stopped. She was put on clobazam, antihistamines, steroids, adequate oral, parenteral hydration, and adequate calories. Other symptomatic management was done accordingly.

**Fig. 1:**
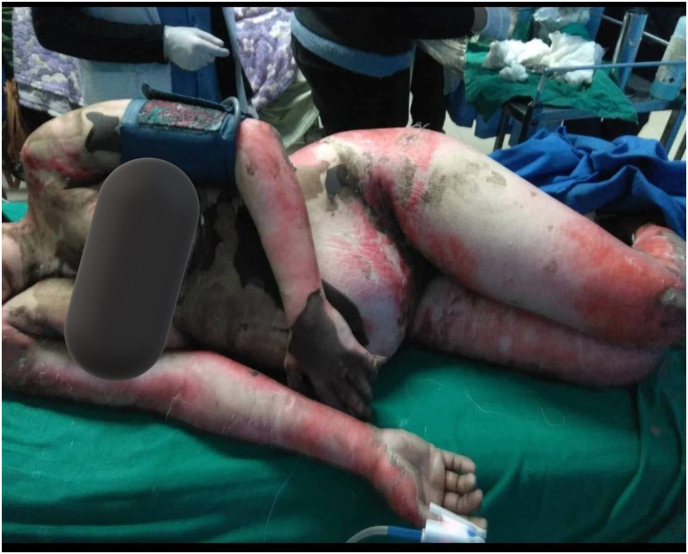
Epidermal peeling involving more than 80% of body surface area with underlying erythema and positive nikolsky sign.

Over the next few days, her lesions and erythema progressed along with epidermal peeling to involve more than 80% of the Body Surface area. She started developing fever, her swab culture showed Methicillin Resistant CoNS, and blood culture showed Acinetobacter spp. She was switched to sensitive antibiotics. Her WBC count decreased to 1520 cells per cubic ml and absolute Neutrophil count to 608. Inj. Filgrastim was started until the absolute neutrophil count rose above 1000. Aminotransferases, SGOT, and SGPT values were also increased to 378 and 78. However, procalcitonin values were within the normal range. She also developed 2 episodes of GTCS which was managed with midazolam and an increasing dose of clobazam (See Fig. 1).

Gradually, her condition started improving. Her fever subsided, counts increased, cultures became negative, seizures controlled and skin lesions started improving. After 9 days of ICU admission, she was shifted to ward than a week later discharged home.

## Discussion

Lamotrigine, a phenyltriazine class of newer antiepileptic chemically differs from aromatic anticonvulsants and so does the incidence of adverse cutaneous manifestation which is around 3–10% with lamotrigine and slightly higher with later [10]. These manifestations are usually noted within 8 weeks of initiation of treatment [11].

Exfoliative dermatitis-like SJS and TEN are both severe, episodic acute mucocutaneous intolerance reactions, characterized by a rapidly expanding erythematous or purplish macular rash, often with target lesions, epidermal necrosis and prominent involvement of more than one mucosal site. TEN is differentiated from SJS by the extent of body surface area involvement. In TEN, more than 30% of the skin area is involved, whereas, in SJS, less than 10% of the skin area is affected [12].

Though these severe cutaneous lesions are associated with higher doses, over-rapid titration, and concomitant use of valporate [6], our case might have differed in this aspect considering the lower initial dose and monotherapy. However, females have a higher risk for antiepileptic skin reactions compared to males; probably due to hormonal factors [13] which again might have influenced our case considering the postpartum status and recent studies have suggested a strong association between HLA-B*1502 and AED-induced SJS/TEN in patients of Asian ethnicity [14]. Though we didn'*t*-test for that allele, these factors also cannot be easily overlooked as a contributing factor in this case. Also, the use of lamotrigine is less in many developing nations which might have also led to the reporting of a lesser number of cases.

The average reported mortality of TEN is 25–35%. The SCORTEN score is the most widely used system to standardize the evaluation of risk and prognosis in patients with TEN [15]. With SCORTEN score of 2, predicted mortality in our patient was 12.1% [8]. So, with prompt supportive Care and below the average predicted mortality, our patient had an eventful but good recovery.

## Learning points

1. Severe exfoliative dermatitis like Steven Johnson syndrome and Toxic Epidermal Necrolysis are less reported with newer antiepileptics like lamotrigine as a monotherapy compared to aromatic anticonvulsants.
2. In a developing country like ours where lamotrigine is not used that frequently compared to other anticonvulsants, the severe adverse cutaneous reaction correlation with the offending drug may be overlooked.
3. Prompt diagnosis, withdrawal of offending agent and proper supportive care can help prevent mortality in such case.

## Ethical approval

This study was conducted in accordance with ethical standard and informed written consent was taken from patient for publication of this case report.

## Sources of funding

There is no any source of funding for this case report.

## Author contribution

1. Kiran Kumar KC wrote the report. And he was directly involved in patient's care during his stay in ICU.
2. Trishant Limbu also wrote the report and revised it with relevant references. And he was directly involved in patient's care during his stay in ICU.
3. Shirish Shakti Maskay worked for literature review and revision of the case report into its final version. He was also directly involved in the patient's care.
4. Subhash Prasad Acharya provided support and mentorship for development, writing and revision of this case report. And he was directly involved in patient's care during his stay in ICU.
5. Anil Bhasima took the history, pictures, performed clinical examinations and was directly involved in patient's care

## Trial registry number

1. Name of the registry:
2. Unique Identifying number or registration ID:
3. Hyperlink to your specific registration (must be publicly accessible and will be checked):

## Guarantor

Kiran Kumar KC.He is the first author and corresponding author for this case report.

## Provenance and peer review

Not commissioned, externally peer reviewed.

## Declaration of competing interest

There is no any conflicts of interest.
